# Characteristics of anti–integrin **α**_v_**β**_6_ autoantibodies in patients with ulcerative colitis

**DOI:** 10.1172/jci.insight.192953

**Published:** 2026-01-08

**Authors:** Ikuhisa Takimoto, Masahiro Shiokawa, Yoshihiro Nishikawa, Takeshi Kuwada, Sakiko Ota, Darryl Joy C. Juntila, Takafumi Yanaidani, Kenji Sawada, Ayako Hirata, Muneji Yasuda, Koki Chikugo, Risa Nakanishi, Masataka Yokode, Yuya Muramoto, Shimpei Matsumoto, Tomoaki Matsumori, Tsutomu Chiba, Hiroshi Seno

**Affiliations:** 1 Department of Gastroenterology and Hepatology, Kyoto University Graduate School of Medicine, Sakyo-ku, Kyoto, Japan.; 2 Kansai Electric Power Hospital, Fukushima-ku, Osaka, Japan.

**Keywords:** Autoimmunity, Gastroenterology, Integrins

## Abstract

Ulcerative colitis (UC) is a chronic inflammatory condition of the colon that primarily affects the mucosal layer. Previously, we identified autoantibodies against integrin α_v_β_6_ in patients with UC. In this study, we established monoclonal antibodies (mAbs) from patients with UC to reveal the features and functions of these anti–integrin α_v_β_6_ autoantibodies. We identified two shared heavy chain complementarity-determining region 3 (CDR3) amino acid sequences among different patients with UC. Notably, several mAbs contained the RGD sequence in their heavy chain CDR3 that mimicked the key recognition sequence of integrin α_v_β_6_ ligands such as fibronectin. Almost all mAbs selectively reacted with integrin α_v_β_6_ in the presence of divalent cations (Ca^2+^ and Mg^2+^) and blocked fibronectin–integrin α_v_β_6_ binding. MAbs that shared the same heavy chain CDR3 amino acid sequence showed differences in reactivity to integrin α_v_β_6_, indicating that the reactivity of these mAbs is also affected by the light chain. Some of the mAbs showed varying degrees of cross-reactivity with integrin α_v_β_3_. The identification of shared CDR3 amino acid sequences in anti–integrin α_v_β_6_ antibodies from several patients with UC suggests a common mechanism underlying their production, which may help elucidate the pathogenesis of UC.

## Abstract 2

Common amino acid sequences in the heavy-chain CDR3 are shared among anti-integrin avb6 autoantibodies of patients with UC.

## Introduction

Ulcerative colitis (UC) is a chronic inflammatory bowel disease marked by continuous mucosal inflammation that begins in the rectum and spreads toward the proximal colon (1–3). The etiology of UC is not yet clearly understood; however, it is hypothesized that genetic predisposition and environmental factors drive immune dysregulation, leading to various grades of epithelial damage along the intestinal tract (1–3).

We previously reported that most patients with UC have autoantibodies targeting integrin α_v_β_6_, and the antibody titer correlates with disease severity; furthermore, IgG from patients with UC inhibits binding between integrin α_v_β_6_ and fibronectin (4). Subsequently, a nationwide multicenter study in Japan confirmed the diagnostic utility of anti–integrin α_v_β_6_ antibodies for UC (5), and several groups have confirmed the presence of anti–integrin α_v_β_6_ antibodies in patients with UC from various countries (6–8). Importantly, a study revealed that these antibodies can be detected up to a decade before a clinical diagnosis of UC, and that elevated antibody titers are linked to poor UC-related prognoses (6). However, the pathophysiological significance of anti–integrin α_v_β_6_ antibodies in UC has not yet been fully elucidated.

Integrins are transmembrane receptors that facilitate interactions between cells and the extracellular matrix (9, 10). Integrin heterodimers are composed of α and β subunits, which are noncovalently linked (9). In mammals, the integrin family comprises 18 α subunits and 8 β subunits, forming a total of 24 distinct heterodimeric combinations (9). Among them, integrin α_v_β_6_ is expressed exclusively on epithelial cells, where it functions as a receptor for extracellular matrix proteins, including fibronectin (10). Additionally, integrin α_v_β_6_ binds to latency-associated protein (LAP) that is in complex with TGF-β, thereby facilitating the activation of TGF-β (10, 11). Thus, integrin α_v_β_6_ plays a pivotal role in maintaining epithelial barrier integrity, protecting against pathogenic infections, and modulating inflammation through TGF-β signaling activation (10, 12–14).

Taking into consideration the correlation between anti–integrin α_v_β_6_ antibody titers and both the severity and outcomes of UC, as well as the critical role of integrin α_v_β_6_ in maintaining colon epithelial integrity, it is likely that this antibody has a substantial impact on UC pathogenesis. Recent studies have also highlighted the importance of B cells in the pathogenesis of UC (15–17). Therefore, we aimed to evaluate the pathophysiological function of anti–integrin α_v_β_6_ antibodies in UC. In this study, we established anti–integrin α_v_β_6_ monoclonal antibodies (mAbs) from the patients with UC and evaluated their characteristics.

## Results

### Anti–integrin α_v_β_6_ antibodies from patients with UC share common CDR3 amino acid sequences.

**Figure 1:**
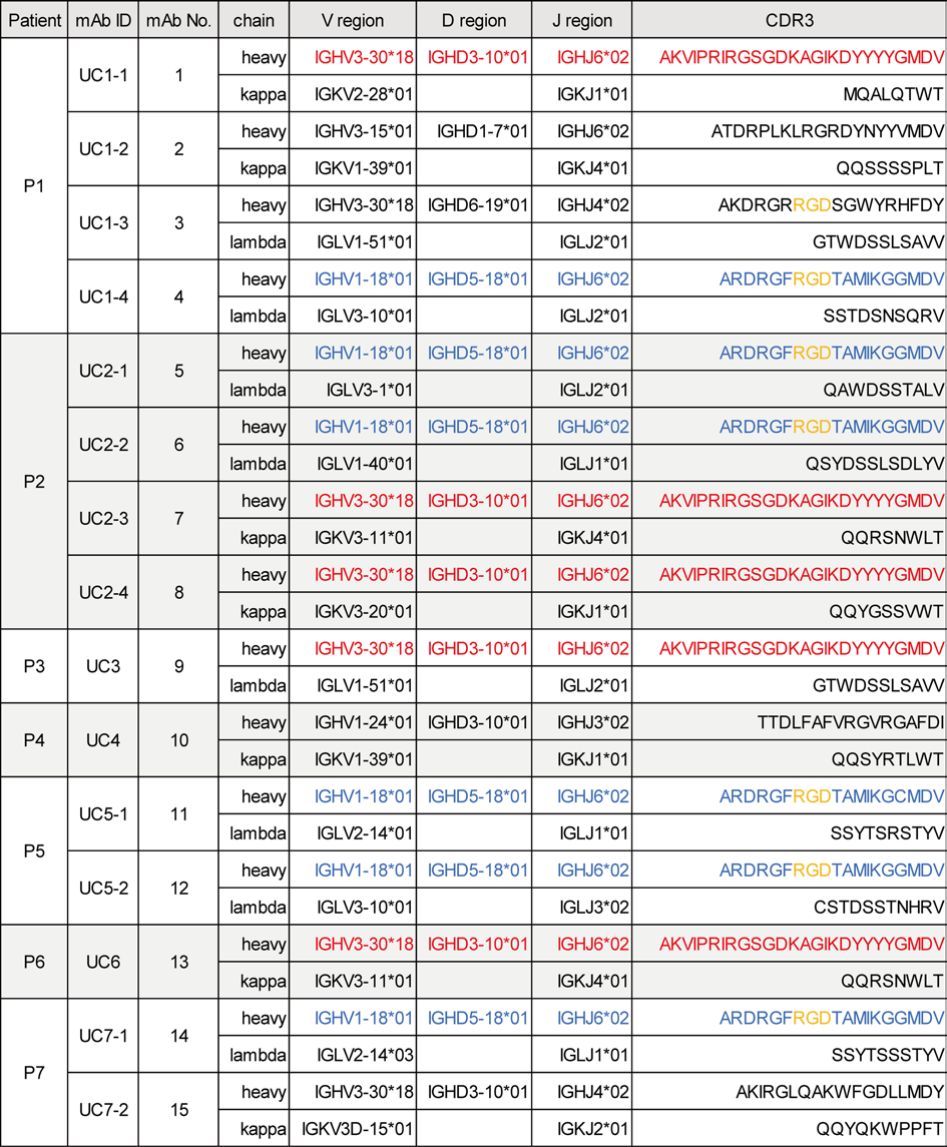
Gene usage and CDR3 amino acid sequence of each mAb. The variable regions of the heavy and light chains of the antibodies were analyzed using IgBLAST. The V, D, and J gene usage, along with the amino acid sequences of the complementarity-determining region 3 (CDR3) for each mAb, is presented.

**Figure 2:**
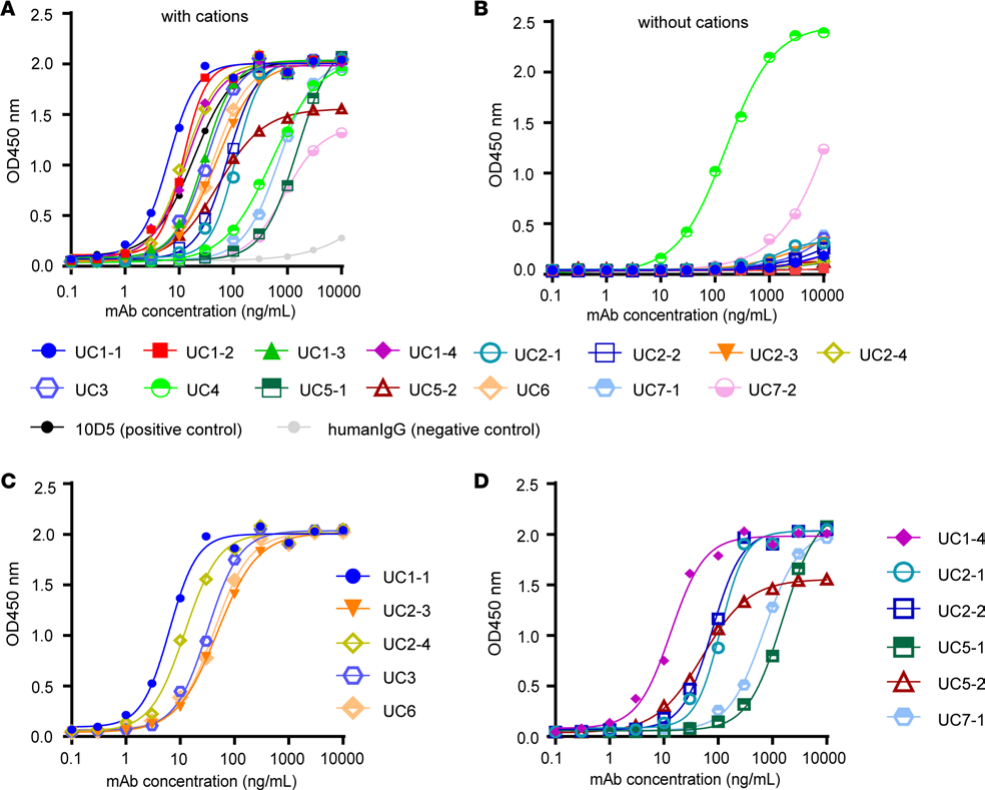
Evaluation of reactivity of each mAb with integrin α_v_β_6_. (**A**) The reactivity of each mAb to integrin α_v_β_6_ was evaluated using ELISA in the presence of Mg^2+^ and Ca^2+^. All mAbs reacted with integrin α_v_β_6_, albeit with different reactivity. (**B**) In ELISA in the absence of Mg^2+^ and Ca^2+^, all mAbs except UC4 lost reactivity to integrin α_v_β_6_. (**C** and **D**) ELISA to estimate the reactivity against integrin α_v_β_6_ of antibodies with a common heavy chain CDR3 amino acid sequence — UC1-1, UC2-3, UC2-4, UC3, and UC6 (carrying the CDR-H1 sequence) (**C**) and UC1-4, UC2-1, UC2-2, UC5-1, UC5-2, and UC7-1 (carrying the CDR-H2 sequence) (**D**).

To investigate the properties of anti–integrin α_v_β_6_ autoantibodies from patients with UC, we initially established 15 anti–integrin α_v_β_6_ mAbs using peripheral blood mononuclear cells (PBMCs) or lymph node samples of 7 patients with UC with anti–integrin α_v_β_6_ antibodies. The clinical characteristics of the patients are summarized in Supplemental Table 1 (supplemental material available online with this article; https://doi.org/10.1172/jci.insight.192953DS1). As shown in Figure 1, the 7 patients were given unique identification numbers, P1 to P7, and the obtained mAbs were given unique identification numbers (UC1-1 to UC7-1) along with serial numbers (nos. 1–15). To characterize each mAb, we analyzed complementarity-determining region 3 (CDR3) sequences, which are crucial for antigen specificity, along with variable region gene usage patterns (Figure 1) and the frequency of somatic hypermutations (SHMs) in the V gene region (Supplemental Table 2). SHM frequency was calculated by counting of the number of mutated sequences against the annotated germline sequences of V region using IgBLAST. Using enzyme-linked immunosorbent assay (ELISA), we confirmed that these mAbs reacted with integrin α_v_β_6_ (Figure 2A).

Notably, we identified 2 distinct CDR3 amino acid sequences of the heavy chain that were shared among some of the mAbs obtained from the 7 patients with UC: one was AKVIPRIRGSGKAGIKDYYYGMDV (CDR-H1), encoded by IGHV3-30*18/IGHD3-10*01/IGHJ6*02 (shown in red in Figure 1); and the other was ARDRGFRGDTAMIKGGMDV (CDR-H2), encoded by IGHV1-18*01/IGHD5-18*01/IGHJ6*02 (shown in blue in Figure 1). MAbs no. 1 (UC1-1), no. 7 (UC2-3), no. 8 (UC2-4), no. 9 (UC3), and no. 13 (UC6) shared the first sequence, whereas mAbs no. 4 (UC1-4), no. 5 (UC2-1), no. 6 (UC2-2), no. 11 (UC5-1), no. 12 (UC5-2), and no. 14 (UC7-1) shared the second sequence. Notably, among the mAbs that shared the second sequence in the heavy chain CDR3, UC5-1 had a one-residue variation.

Interestingly, some mAbs had RGD sequences (shown in yellow in Figure 1) in the CDR3 region of the heavy chain. Integrin α_v_β_6_ recognizes RGD sequences of its physiological ligands, including fibronectin and LAP (9). The presence of RGD sequences in the CDR3 regions of these mAbs suggests that they compete with the ligands (e.g., fibronectin and LAP) for binding to integrin α_v_β_6_.

Analysis of CDR3 amino acid lengths and SHM frequencies of the V region gene (Supplemental Table 2) showed that these mAbs had longer CDR3 regions and lower SHM rates in the V region gene than do those reported in healthy individuals (7.47%) (18). Overall SHM frequencies were low; however, mutations were enriched in the CDRs, with higher rates than those in the framework regions (FR) (Supplemental Figure 1A). Amino acid mutations followed a similar distribution (Supplemental Figure 1B).

Most antibodies with CDR-H1 or CDR-H2 carried only 1–3 replacement mutations in the heavy chain CDRs and lacked silent mutations (Supplemental Figure 1C). In the FR regions, antibodies with CDR-H1 tended to show lower replacement/silent ratios, while those with CDR-H2 often showed few replacement mutations and no silent ones (Supplemental Figure 1C). These findings suggest that selective introduction of minimal but functional mutations may be sufficient for antigen binding, and that FR mutations may also contribute to binding in antibodies containing CDR-H2.

SHM levels in light chains varied widely. Some antibodies had heavily mutated CDRs, whereas others retained entirely germline light chains. Notably, several antibodies showed integrin α_v_β_6_ reactivity without any light chain mutations, suggesting that such mutations are not always required for binding.

Taken together, these findings suggest that B cells using germline VH genes with intrinsic autoreactive potential acquire or enhance integrin α_v_β_6_ binding through limited, functionally targeted replacement mutations.

### All mAbs except UC4 react against integrin α_v_β_6_ in a cation-dependent manner.

Reactivity and affinity of each mAb to integrin α_v_β_6_ were assessed using ELISA and biolayer interferometry (BLI), respectively. To validate results obtained in ELISA, we used the mouse anti–human integrin α_v_β_6_ antibody 10D5, which is known to inhibit both fibronectin and LAP binding (19), as a positive control, and IgG purified from healthy individuals as negative controls. The reactivity of all the mAbs against integrin α_v_β_6_ was confirmed using ELISA (Figure 2A), and the half-maximal effective concentration (EC_50_) values of the mAbs were calculated (6.30–1,537 ng/mL) (Table 1).

In our previous study, we showed that the serum titer of anti–integrin α_v_β_6_ antibodies in patients with UC increased significantly with the addition of Ca^2+^ and Mg^2+^ (4). Therefore, we compared the binding of mAbs to integrin α_v_β_6_ in the presence and absence of Ca^2+^ and Mg^2+^. The mAbs except UC4 showed marked loss of reactivity in ELISA in the absence of Ca^2+^ and Mg^2+^ (Figure 2B). Ca^2+^ and Mg^2+^ are important for integrin heterodimer formation, activation of integrins, and binding to its ligands (20–22). This cation-dependent binding suggests that these antibodies recognize integrin α_v_β_6_ in its active conformation maintained by divalent cations. In other words, these mAbs were directed toward active integrin α_v_β_6_. On the other hand, only UC4 reacted with integrin α_v_β_6_ in a cation-independent manner, indicating that its binding site is different from that of other mAbs.

To investigate differences in reactivity with integrin α_v_β_6_ caused by differences in light chains, we chose mAbs with a common CDR3 amino acid sequence in the heavy chain (Figure 2C: CDR-H1; and Figure 2D: CDR-H2) but with different light chains and analyzed their reactivities. Notably, UC2-3 and UC6 had identical CDR3 amino acid sequences in the light chain, and the light chain CDR3 of UC5-1 and UC7-1 had highly similar amino acid sequences. The other antibodies with common heavy chain CDR3 amino acid sequences had different light chain CDR3 amino acid sequences (UC1-1, UC2-4, and UC3; UC1-4, UC2-1, UC2-2, and UC5-2). UC2-3 and UC6 as well as UC5-1 and UC7-1 showed similar reactivities, consistent with sequence similarity; however, the mAbs with common heavy chain CDR3 amino acid sequences but different light chain amino acid sequences showed different reactivities in ELISA (Figure 2, C and D), suggesting that the light chain amino acid sequence is also important for determining binding specificity to integrin α_v_β_6_.

Next, the affinity of each mAb to integrin α_v_β_6_ was measured using BLI (Table 2 and Supplemental Figure 2A). First, we conducted BLI in the presence of cations in the buffer. Most mAbs had sufficient affinity for integrin α_v_β_6_, similar to the results seen in ELISA; however, some of the mAbs that had shown high EC_50_ values (UC5-1, UC7-1, and UC7-2) in ELISA showed low affinity in BLI. In particular, UC4 did not bind sufficiently to integrin α_v_β_6_ in BLI. To further characterize the binding properties of UC4, which showed unique cation-independent reactivity in ELISA, we performed BLI measurements using both UC4 and UC1-1 in the absence of cations (Supplemental Figure 2, B and C). We used UC1-1 as a representative cation-dependent antibody. In BLI measurements without cations, neither antibody bound to integrin α_v_β_6_. Notably, UC4 showed no binding in BLI even in the presence of cations, despite showing reactivity in ELISA. The reason for this discrepancy remains to be determined.

### Effect of mAbs on integrin α_v_β_6_–fibronectin and integrin α_v_β_6_–LAP binding.

**Figure 3:**
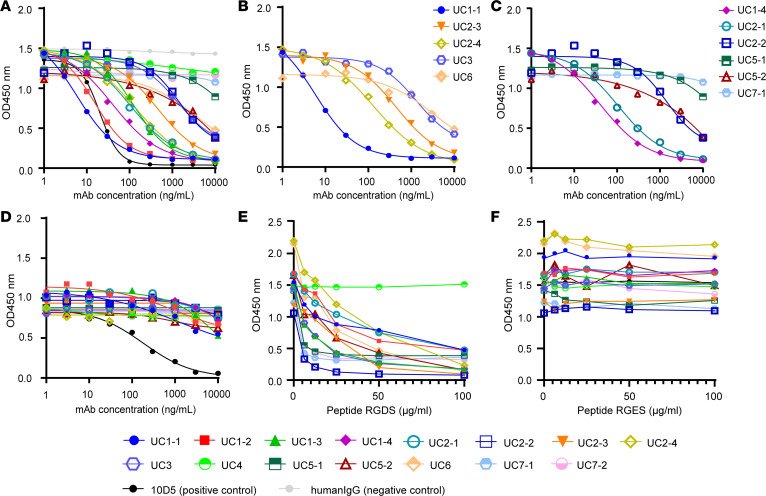
Blocking of integrin α_v_β_6_–fibronectin binding or integrin α_v_β_6_–LAP binding by each mAb in ELISA. (**A**) Blocking activity of each mAb on integrin α_v_β_6_–fibronectin binding was evaluated using a solid-phase integrin α_v_β_6_ binding assay. MAbs with low EC_50_ blocked integrin α_v_β_6_–fibronectin binding in a concentration-dependent manner. MAbs with high EC_50_ concentrations (UC4, UC5-1, UC7-1, and UC7-2) showed little blocking activity. UC2-2, UC5-2, and UC6 did not have sufficient blocking activity to reach a plateau within the measured concentration range. (**B** and **C**) Blocking activity on integrin α_v_β_6_–fibronectin binding was compared between antibodies with a common amino acid sequence in the heavy chain CDR3 — UC1-1, UC2-3, UC2-4, UC3, and UC6 (carrying the CDR-H1 sequence) (**B**) and UC1-4, UC2-1, UC2-2, UC5-1, UC5-2, and UC7-1 (carrying the CDR-H2 sequence) (**C**). (**D**) Blocking activity of each mAb against integrin α_v_β_6_–LAP binding was evaluated using the same method. (**E** and **F**) Effect of RGDS peptides (**E**) and control RGES peptides (**F**) at various concentrations on the binding of mAbs to integrin α_v_β_6_. LAP, latency-associated protein.

To investigate the function of each mAb, we examined blocking activities of the mAbs on integrin α_v_β_6_–fibronectin and integrin α_v_β_6_–LAP binding. Both fibronectin and LAP bind to the RGD binding site of integrin α_v_β_6_ via their RGD motifs (9). In a solid-phase binding assay, 8 of the 15 mAbs firmly blocked integrin α_v_β_6_–fibronectin binding in a dose-dependent manner (Figure 3A). The calculated half-maximal inhibitory concentration (IC_50_) values are shown in Table 3. However, some mAbs with high EC_50_ values (UC4, UC5-1, UC7-1, and UC7-2) showed very limited blocking activity. UC2-2, UC5-2, and UC6 did not have sufficient blocking activity to reach a plateau within the measured concentration range, and the IC_50_ could not be calculated. Notably, mAbs with common heavy chain CDR3 amino acid sequences showed varying degrees of blocking activity (Figure 3, B and C). Only marginal inhibition of integrin α_v_β_6_–LAP binding was observed for the patient-derived mAbs (Figure 3D), in contrast to the clear blocking of integrin α_v_β_6_–fibronectin binding. To better understand this differential blocking effect, we compared the affinities of fibronectin and LAP to integrin α_v_β_6_ using BLI (Supplemental Figure 3, A and B, and Supplemental Table 3). Fibronectin showed measurable binding kinetics (dissociation constant [K_D_] = 47.18 nM), whereas LAP demonstrated extremely stable binding with negligible dissociation (dissociation rate constant [K_off_] < 1.0 × 10^–7^ 1/s); this indicated a substantially higher affinity to integrin α_v_β_6_. The control antibody 10D5 demonstrated much higher affinity for integrin α_v_β_6_ (Supplemental Figure 3C) and was able to effectively block LAP binding. These results suggest that the affinity of patient-derived mAbs for integrin α_v_β_6_ is not sufficient to overcome the strong binding of LAP, thereby limiting their ability to inhibit integrin α_v_β_6_–LAP interaction.

Integrin α_v_β_6_ binds to the RGD sequence in its ligands (9). Based on our results and those of a previous study (4), we hypothesized that the mAbs interact with the RGD binding site in integrin α_v_β_6_. Therefore, we evaluated whether RGDS peptides could inhibit the binding of each of the studied mAbs with integrin α_v_β_6_. In line with a previous report (4), RGDS peptides inhibited the binding of all mAbs, except that of UC4, to integrin α_v_β_6_ in a dose-dependent manner (Figure 3E), whereas control RGES peptides did not show such effects (Figure 3F). The binding of UC4 with integrin α_v_β_6_, which showed reactivity with integrin α_v_β_6_ in the absence of cations in ELISA, was inhibited by neither RGDS nor RGES, indicating that this antibody reacts with integrin α_v_β_6_ in an RGD-independent manner. The other mAbs were suggested to be critically dependent on the RGD binding site for binding to integrin α_v_β_6_.

### Cross-reactivity of anti–integrin α_v_β_6_ antibodies with integrin α_v_β_3_.

**Figure 4:**
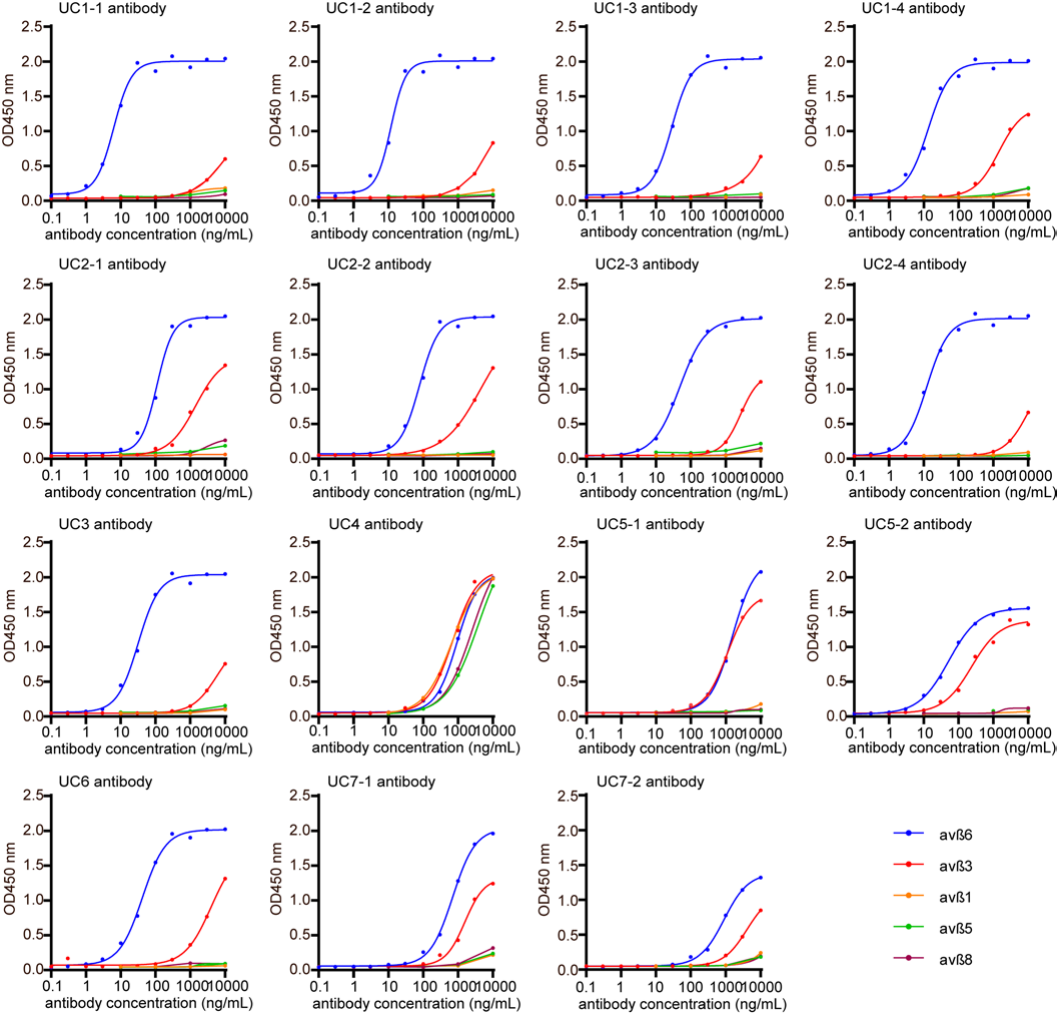
Cross-reaction of mAbs to other integrins. Using ELISA, each mAb was tested to evaluate whether it reacted with integrin α_v_β_1_, α_v_β_3_, α_v_β_5_, and α_v_β_8_.

Previously, we reported that sera of certain patients with UC contained antibodies against both integrin α_v_β_6_ and α_v_β_3_, with anti-α_v_β_3_ antibody titers being consistently lower than those of anti-α_v_β_6_ antibodies (4). However, it remained unclear whether anti–integrin α_v_β_3_ antibodies existed independently or whether anti–integrin α_v_β_6_ antibodies cross-reacted with integrin α_v_β_3_. Therefore, using ELISA, we tested whether each of the mAbs used in this study reacts with integrin α_v_β_1_, α_v_β_3_, α_v_β_5_, and α_v_β_8_, all of which have α_v_ chains and recognize RGD peptides in their physiological ligands (9) (Figure 4). UC4 showed almost identical reactivity to all integrins used for testing. Therefore, this antibody might react with either the α_v_ chain or a common region of the β chain, such as the β tail domain. UC5-1 and UC5-2 showed reactivity not only to integrin α_v_β_6_ but also to integrin α_v_β_3_. Other mAbs reacted slightly to integrin α_v_β_3_ at high concentrations and had no notable reaction to other integrins. These results suggested that anti–integrin α_v_β_6_ antibodies potentially cross-react with integrin α_v_β_3_.

### IgG and mAbs derived from patients with UC exhibit similar blocking activity on integrin α_v_β_6_–fibronectin/LAP binding.

**Figure 5:**
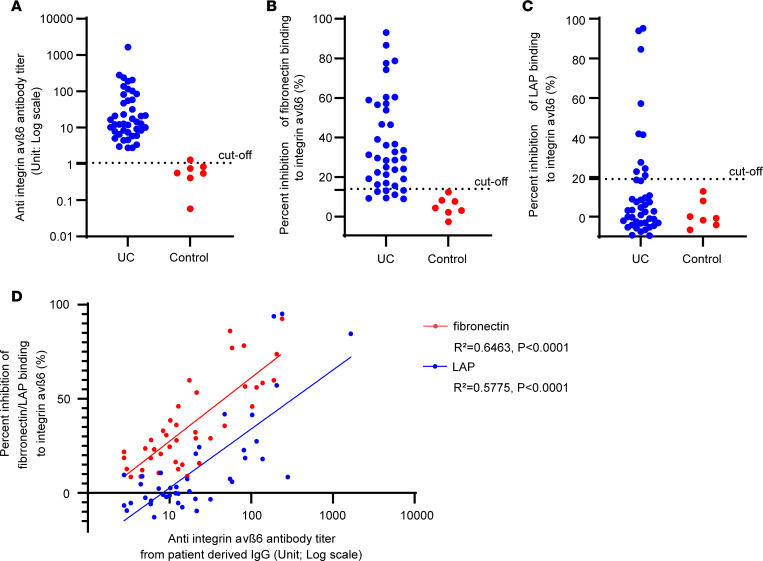
Evaluation of anti–integrin α_v_β_6_ antibodies using IgG from patients with UC. (**A**) Presence of anti–integrin α_v_β_6_ antibodies in IgG derived from patients with UC. (**B** and **C**) Inhibitory effect of patient-derived IgG on integrin α_v_β_6_–fibronectin binding (**B**) and integrin α_v_β_6_–LAP binding (**C**). (**D**) Correlation of percentage inhibition of integrin α_v_β_6_–fibronectin binding (**B**) and integrin α_v_β_6_–LAP binding with antibody titers (fibronectin: *R*^2^ = 0.6463, *P* < 0.0001; LAP: *R*^2^ = 0.5775, *P* < 0.0001).

We previously reported that IgG derived from patients with UC blocked integrin α_v_β_6_–fibronectin binding (4); however, we had not tested whether it blocks integrin α_v_β_6_–LAP binding. Using the IgG derived from 42 patients with UC who were part of the training group of our previous study and IgG from 8 control patients (4), we evaluated anti–integrin α_v_β_6_ IgG titer of patients with UC, and then evaluated blocking activity of these IgG samples on integrin α_v_β_6_–fibronectin and –LAP binding (Figure 5, A–C, and Supplemental Table 4).

Consistent with our previous results (4), anti–integrin α_v_β_6_ IgG titers were positive in 100% of cases (42 of 42) and showed a substantial inhibitory effect on integrin α_v_β_6_–fibronectin binding in 76.2% of cases (32 of 42). Further, 23.8% cases (10 of 42) showed a substantial inhibitory effect on integrin α_v_β_6_–LAP binding. Both inhibitory effects were correlated with antibody titers (Figure 5D). The patient-derived IgG samples showed blocking activity similar to that of the previously evaluated mAbs, based on their ability to inhibit integrin α_v_β_6_–fibronectin and integrin α_v_β_6_–LAP binding. In both cases, inhibition of integrin α_v_β_6_–fibronectin binding was stronger than that of integrin α_v_β_6_–LAP binding. This similarity suggests that these mAbs capture key features of anti–integrin α_v_β_6_ antibodies in patients with UC.

In contrast to the mAbs, a small proportion of IgG samples from patients exhibited a strong inhibitory effect on integrin α_v_β_6_–LAP binding. It is possible that these individuals possess high-affinity anti–integrin α_v_β antibodies, comparable to the 10D5 antibody. However, given that the majority of IgG samples showed limited or no inhibition of integrin α_v_β_6_–LAP binding, such high-affinity antibodies appear to be relatively uncommon and are unlikely to constitute the predominant anti–integrin α_v_β_6_ antibody population in patients with UC.

## Discussion

In this study, we established 15 anti–integrin α_v_β_6_ mAbs from PBMCs or lymph node samples of patients with UC. We identified 2 predominant CDR3 amino acid sequences in the heavy chain that were shared among different patients. One sequence was encoded by IGHV3-30*18/IGHD3-10*01/IGHJ6*02 and the other by IGHV1-18*01/IGHD5-18*01/IGHJ6*02. Almost all mAbs reacted with integrin α_v_β_6_ in the presence, but not in the absence, of cations. These mAbs inhibited integrin α_v_β_6_–fibronectin binding, but showed substantially lower inhibitory effect against integrin α_v_β_6_–LAP binding. The binding of mAbs with integrin α_v_β_6_ was inhibited by RGDS peptide. Notably, several mAbs contained the RGD sequence in their heavy chain CDR3. Taken together, these data suggest that these mAbs bind to integrin α_v_β_6_ in an RGD site–dependent manner. Evaluation via ELISA revealed that some of the anti–integrin α_v_β_6_ mAbs cross-reacted against integrin α_v_β_3_. The functional properties of these mAbs are similar to those observed in IgG samples, suggesting that these mAbs capture key features of anti–integrin α_v_β_6_ antibodies in UC.

Our analysis revealed that 2 distinct CDR3 amino acid sequences were particularly prevalent among different patients with UC. The first sequence, encoded by IGHV3-30*18/IGHD3-10*01/IGHJ6*02, was found in 5 mAbs from 4 patients, whereas the second sequence, encoded by IGHV1-18*01/IGHD5-18*01/IGHJ6*02, was shared among 6 mAbs from 4 patients. The presence of such shared heavy chain CDR3 amino acid sequences has been observed in other autoimmune conditions, including anti-desmoglein antibodies in patients with pemphigus (23, 24) and anti-ADAMTS13 antibodies in those with thrombotic thrombocytopenic purpura (25). Indeed, it is increasingly recognized that patients with diverse genetic backgrounds and immunological histories produce stereotyped B cell receptors (BCRs) in response to a specific antigen (26). The identification of these stereotyped anti–integrin α_v_β_6_ antibodies in patients with UC suggests common humoral immune mechanisms underlying the production of these antibodies.

Our antibodies exhibited low SHM levels, often with a few replacement mutations in the CDRs, yet retained antigen reactivity. This is in line with recent findings suggesting that antibody affinity can be optimized not through extensive mutation, but via selective, minimal functional changes to germline VH genes (27). According to a previous report, in plasma cells in the intestinal tract of patients with UC, the IGHJ6 usage is increased, CDR3 length is longer, and the SHM frequency is lower than in healthy controls (17). In this study, most mAbs were found to have a long CDR3; in particular, mAbs encoded by IGHV3-30*18/IGHD3-10*01/IGHJ6*02 had many features, such as increased IGHJ6 usage and low SHM rate, that match earlier reports regarding characteristics of antibodies in patients with UC. The agreement between the current results and those of previous studies (17) may reflect the widespread sharing of these mAbs among patients with UC. The mechanisms of production of these mAbs and the roles of such mAbs in the pathology of UC are yet unknown and require further research.

The mAbs established in this study shared many characteristics with antibodies present in the sera or with IgG from patients with UC who were positive for anti–integrin α_v_β_6_ antibodies (4). Furthermore, some new insights were also gained. Notably, the mAbs in this study comprised both cation-dependent and cation-independent antibodies. The cation-dependent antibodies were expected to bind via the RGD binding site in integrin α_v_β_6_. In contrast, UC4 was the unique antibody that reacted with integrin α_v_β_6_ in a cation-independent manner, with evidence suggesting that it may be reacting with integrin monomers independent of the RGD binding site.

We observed in this study that some anti–integrin α_v_β_6_ mAbs cross-reacted with integrin α_v_β_3_. We have reported earlier that some patients with UC have antibodies that react with integrin α_v_β_3_ (4). Although we cannot ignore the possibility that anti–integrin α_v_β_3_–specific autoantibodies coexist independent of anti–integrin α_v_β_6_ autoantibodies in the sera of patients with UC, our current findings suggest that the previously observed reactivity to integrin α_v_β_3_ of the sera of patients with UC may be explained by cross-reactivity of the anti–integrin α_v_β_6_ antibodies to integrin α_v_β_3_.

Using both mAbs and patient-derived IgG samples, we found that inhibition of integrin α_v_β_6_–LAP binding was consistently weaker than that of fibronectin binding. This likely reflects the stronger affinity of LAP for integrin α_v_β_6_, as shown by our BLI analysis. Accordingly, most mAbs exhibited only limited ability to interfere with LAP binding. Interestingly, a subset of patient IgG samples demonstrated appreciable inhibition of LAP binding; this suggested that high-affinity anti-α_v_β_6_ antibodies are present in a fraction of patients, although they do not appear to represent the predominant anti–integrin α_v_β_6_ antibody population in UC. While the inhibition of fibronectin binding was common, LAP inhibition was generally modest. The in vivo relevance of either effect remains unclear and warrants further investigation.

The major limitation of this study is that both the number of mAbs and the number of patients from whom those mAbs were obtained were rather small. Moreover, patient-derived anti–integrin α_v_β_6_ antibodies were randomly collected; therefore, the results may not reveal the complete picture of anti–integrin α_v_β_6_ antibodies in patients with UC. Given this limited dataset, the broad representativeness of the identified shared CDR3 sequences across patients with UC still remains to be further examined. Experiments using samples from a larger number of patients with UC will be necessary to prove universality of the characteristics of anti–integrin α_v_β_6_ antibodies. In addition, we have not been able to examine whether these anti–integrin α_v_β_6_ antibodies are directly involved in the pathogenesis of UC. In particular, the presence of an RGD motif or the ability to recognize the RGD binding site may enhance the functional impact of certain anti-α_v_β_6_ antibodies by interfering with ligand binding or downstream signaling. Further studies using in vivo models are needed to clarify whether such antibodies contribute distinctively to UC pathogenesis. Classification based on these features may offer new insights into disease mechanisms. Furthermore, we obtained mAbs only at one point for each patient; therefore, sequential samples from the same patient were lacking. Longitudinal studies with serial sampling from a larger number of patients are warranted to explore potential changes in the qualitative and functional characteristics of anti–integrin α_v_β_6_ antibodies during the disease course in individual patients, and to provide important insights into their roles in disease onset and exacerbation.

In conclusion, the mAbs established from patients with UC mainly recognized integrin α_v_β_6_ in its active conformation. This study identified 2 predominant CDR3 amino acid sequences in the heavy chain of mAbs. Most mAbs demonstrated cation-dependent binding to integrin α_v_β_6_ and selective inhibition of integrin α_v_β_6_–fibronectin binding. The functional properties of these mAbs are similar to those observed in IgG samples from 42 patients with UC; this suggests that these mAbs capture key features of anti–integrin α_v_β_6_ antibodies in UC. Taken together, these data indicate that anti–integrin α_v_β_6_ antibodies are deeply involved in the pathophysiology of UC, and these results may provide potential leads for the development of new therapeutic strategies. As this study characterized a limited number of mAbs, further investigation with larger antibody panels will be needed to fully understand the diversity and clinical significance of these autoantibodies.

## Methods

### Sex as a biological variable.

This study included samples from 4 male and 3 female patients with UC. Sex was not considered a biological variable in the analyses because the study focused on the molecular and functional characterization of anti–integrin α_v_β_6_ antibodies, which do not show sex-related differences. Therefore, the findings are expected to be relevant to both sexes.

### Study design.

The purpose of this study was to generate and characterize patient-derived anti–integrin α_v_β_6_ antibodies. We used PBMC or mesenteric lymph node samples from patients with UC to identify B cells expressing anti–integrin α_v_β_6_ antibody by B cell immortalization or single-cell sorting using flow cytometry. The gene of the variable region of the antibody was sequenced to produce an anti–integrin α_v_β_6_ mAb. These sequences were annotated using IgBLAST (28) to identify gene usage and amino acid sequences. Based on our previous reports (4), we performed experiments such as ELISA and solid-phase integrin α_v_β_6_ binding assay to characterize the antibodies.

### Patients.

A total of 7 patients with UC undergoing treatment at Kyoto University Hospital participated in this study. The clinical characteristics of the patients are summarized in Supplemental Table 1. Diagnosis of patients was based on a combination of their symptoms, endoscopic findings, histological features, and the lack of alternative diagnoses (29, 30). Patients were considered eligible if they were positive for anti–integrin α_v_β_6_ antibodies, regardless of clinical severity or treatment. The study was conducted in accordance with the Declaration of Helsinki and was authorized by the Ethics Committee of the Graduate School of Medicine, Kyoto University. Written informed consent was obtained from all patients after an explanation of the nature and the possible outcomes of the study. Sample sources (lymph nodes or PBMCs) were selected solely on the basis of clinical availability and procedural feasibility. Lymph node samples were obtained from patients undergoing surgical procedures, which facilitated the collection of a sufficient number of B cells. PBMC samples were obtained from patients who did not undergo surgery. Each patient contributed only one type of sample. No notable differences were observed in the characteristics of antibodies derived from PBMCs and those derived from lymph nodes.

### Sample preparation.

To generate mAbs, blood or lymph node samples were collected from the patients. PBMCs were isolated from whole blood by gradient centrifugation using BD Vacutainer CPT (Becton, Dickinson and Co.). The lymph node samples were obtained from the mesenteric lymph nodes attached to the surgical specimen during total colectomy. Lymph node samples were chopped with a clean scalpel, mashed on a 70 μm mesh using a plunger, and washed with PBS. After centrifugation of the cell suspension at 300*g* for 5 minutes, the supernatant was removed, and 1× RBC Lysis Buffer (pluriSelect) was added. After 3 minutes, the reaction was stopped by addition of PBS with 0.1% BSA. The resulting cell suspension was subjected to centrifugation at 300*g* for 5 minutes to obtain lymph node–derived cells, which were preserved at –80°C for further use.

### Preparation of Epstein-Barr virus reagent.

The Epstein-Barr virus (EBV) reagent was prepared and stocked as a culture supernatant harvested from the B95-8 marmoset cell line obtained from the Japanese Collection of Research Bioresources Cell Bank (cell ID: JCRB9123). B95-8 cells were cultured in RPMI 1640 medium (Fujifilm Wako Pure Chemical) containing 10% FBS at 37°C in 5% CO_2_ until reaching confluence. The culture medium was centrifuged at 400*g* for 10 minutes to remove all residue, and the supernatant was divided into aliquots and stored at –80°C until used for transduction.

### Establishment of lymphoblastoid cell lines by EBV transduction.

Lymphoblastoid cell lines (LCLs) were established as previously reported (31, 32). Briefly, IgM^+^ B cells were removed from PBMCs using magnetic cell sorting with anti-human IgM MicroBeads following the manufacturer’s instructions (Miltenyi Biotec). PBMCs obtained after removal of IgM^+^ B cells were suspended in EBV stock (1 × 10^7^ cells/mL) containing 2.5 μg/mL ODN2006 (Alpha Diagnostic International) and kept at 37°C under 5% CO_2_ for 1 hour. The cells were suspended in RPMI 1640 (2 × 10^5^ cells/mL) containing 20% FBS, streptomycin/penicillin, 2.5 μg/mL ODN2006 CpG, 50 IU recombinant human IL-2 (R&D Systems), and 500 ng/mL cyclosporin A (Tokyo Chemical Industry Co) and seeded at 200 μL/well in round-bottomed 96-well plates. After culture for 2 weeks, ELISA was performed using culture supernatants to identify LCLs producing anti–integrin α_v_β_6_ antibodies, and RNA isolation was performed from these LCLs. Total RNA was extracted from LCLs using an RNeasy Mini Kit (catalog 74106, QIAGEN) following the manufacturer’s protocol.

An alternative method was to scale up the LCLs producing the antibody of interest and then use a cell array method to identify single cells producing anti–integrin α_v_β_6_ antibodies. This method was supported by EVEC Inc. Briefly, antigen-immobilized microbeads were seeded onto microarray chips together with LCLs and allowed to react for several hours. Array chips were washed and stained with anti-human IgG-R-phycoerythrin, and wells with positive beads were identified using fluorescence microscopy. RNA isolation was performed from cells in positive wells.

### Single-cell sorting.

MAbs were generated from antigen-specific B cells using a single-cell sorting protocol based on a previously reported method (33), with minor modifications as detailed below. PBMCs or lymph node cells were resuspended at 1 × 10^6^ cells per 100 μL in FACS buffer, which comprised 49 mL of Dulbecco’s PBS and 1 mL of FBS. Cells were stained on ice for 20 minutes with the following antibodies: Alexa Fluor 700–mouse anti–human CD20 (1:80; clone L27; catalog 560631, Becton, Dickinson and Co.), APC–mouse anti-human IgG (1:20; clone G18-145; catalog 550931, Becton, Dickinson and Co.), and DAPI (1:100; Thermo Fisher Scientific).

To detect integrin α_v_β_6_–specific B cells, a bait complex was prepared by preincubation of biotinylated integrin α_v_β_6_ (IT6-H82E4, ACROBiosystems) with phycoerythrin-conjugated (PE-conjugated) NeutrAvidin (Thermo Fisher Scientific) in FACS buffer at a fixed volumetric ratio (31:1:18 μL, respectively). This bait complex was applied at an approximate cell-to-bait ratio of 20:1. It enables the detection of integrin α_v_β_6_–specific B cells through fluorescent signal amplification. Appropriate negative controls were included to assess background staining. The gating strategy for detection of integrin α_v_β_6_–specific B cells using PBMCs or lymph node cells by flow cytometry is shown in Supplemental Figure 4.

Before sorting, each well of a 96-well PCR plate was preloaded with 4 μL of sorting buffer, comprising 3.1 μL of nuclease-free H_2_O, 0.2 μL of RNasin (40 U/μL; Promega), 0.1 μL of RNaseOUT (40 U/μL; Thermo Fisher Scientific), 0.2 μL of 10× PBS (resulting in 0.5× PBS), and 0.4 μL of 100 mM DTT (final concentration 10 mM; Thermo Fisher Scientific). Single viable B cells (CD20^+^, IgG^+^, DAPI^–^, and PE^+^ for α_v_β_6_ binding) were individually sorted into the wells using a BD FACSAria II cell sorter (Becton, Dickinson and Co.). After sorting, the plates were immediately frozen and stored at –80°C until RNA extraction.

### Single-cell cDNA synthesis and PCR for amplifying variable regions of BCRs.

To characterize the antibody repertoire at the single-cell level, reverse transcription (RT) and semi-nested PCR were performed to amplify the variable regions of Ig heavy and light chain genes from individual B cells.

Sorted single B cells were thawed on ice and first incubated at 65°C for 2.5 minutes with a random-hexamer primer mix comprising 5.6 μL of nuclease-free H_2_O, 0.75 μL of random-hexamer primers (200 ng/μL; Thermo Fisher Scientific), 0.5 μL of NP-40 (10%; Thermo Fisher Scientific), and 0.15 μL of RNaseOUT (40 U/μL; Thermo Fisher Scientific). After incubation, the samples were placed on ice for at least 2 minutes.

Subsequently, an RT mix containing 2.05 μL of nuclease-free H_2_O, 3 μL of 5× RT buffer (Thermo Fisher Scientific), 0.5 μL of dNTP mix (25 mM; Thermo Fisher Scientific), 1 μL of DTT (100 mM), 0.1 μL of RNasin (40 U/μL; Promega), 0.1 μL of RNaseOUT, and 0.25 μL of SuperScript IV reverse transcriptase (200 U/μL; Thermo Fisher Scientific) was added to each well (final volume of RT mix: 7 μL). The RT reaction was performed using the following thermal protocol: 42°C for 10 minutes, 25°C for 10 minutes, 50°C for 10 minutes, and 94°C for 5 minutes, followed by a hold at 4°C. Following RT, the resulting cDNA was diluted with 16 μL of nuclease-free water before PCR amplification.

The variable regions of Ig heavy and light chains were amplified using a semi-nested PCR strategy with Platinum Taq DNA Polymerase or Platinum Taq Green Hot Start DNA Polymerase (Thermo Fisher Scientific), using exactly the same optimized primer set described in the previous study (33), which was specifically designed for efficient amplification of Ig variable regions.

For both the first- and second-round PCRs, reactions were conducted in a 25 μL total volume using Platinum Taq DNA Polymerase (Thermo Fisher Scientific). The master mix comprised the following components per reaction: 14.68 μL of nuclease-free H_2_O, 2.05 μL of 10× Platinum Taq PCR buffer, 1.23 μL of KB Extender (6%), 0.61 μL of MgCl_2_ (50 mM), 0.16 μL of dNTP mix (25 mM), and 0.09 μL each of forward and reverse primers (50 μM), using the same primer sets previously optimized and described in detail (33). For the first-round PCR, 6 μL of diluted cDNA was used as a template; subsequently, 1 μL of the first-round PCR product was used as the template in the second-round PCR. For second-round PCR, Platinum Taq Green Hot Start DNA Polymerase (Thermo Fisher Scientific) was used to enable direct visualization of PCR products on agarose gels via the included tracking dye.

The first-round PCR was performed under the following conditions: 94°C for 2 minutes, followed by 50 cycles at 94°C for 30 seconds, 57°C for 30 seconds, and 72°C for 55 seconds. The second-round PCR used similar conditions, with an extended step involving 72°C for 45 seconds. Second-round PCR products were analyzed by agarose gel electrophoresis, and samples of the correct size (approximately 500 bp for heavy chains and 450 bp for light chains) were subjected to Sanger sequencing. The same protocol was applied to RNA derived from LCLs.

### Analysis of the V region gene sequence of BCRs.

Aliquots of the second-round PCR products were analyzed by 2% agarose gel electrophoresis to confirm the presence of amplicons of the expected size — approximately 500 bp for heavy chain and 450 bp for light chain variable regions. PCR products of the correct size were subjected to Sanger sequencing, and the resulting sequences were annotated using IgBLAST (28) with the IMGT reference database to determine V(D)J gene usage, CDR3 sequences, and repertoire characteristics and to infer clonal relationships. Sequences containing stop codons or out-of-frame rearrangements (i.e., nonproductive sequences) were excluded from further analysis.

### Cloning of the V region gene sequence of BCRs.

For cloning of BCR variable regions, the first-round PCR product was reamplified using KOD -Plus- Neo polymerase (TOYOBO) and the same optimized primer set with vector-compatible overhangs as described in a previous report (33). PCR comprised the following thermal cycle: 98°C for 30 seconds; 35 cycles of 98°C for 10 seconds, 65°C for 30 seconds, and 72°C for 30 seconds; and 72°C for 2 minutes. Before cloning, amplified PCR products were purified using a PCR purification kit (QIAquick PCR Purification Kit, QIAGEN).

AbVec2.0-IGHG1 (IgG1) (Addgene plasmid 80795), AbVec1.1-IGKC (Igk) (Addgene plasmid 80796), and AbVec1.1-IGLC2-XhoI (Igl) (Addgene plasmid 99575) were used as human antibody expression vectors as previously reported (33). EcoRI and SalI were used for IgG1, EcoRI and BsiWI for IgK, and EcoRI and XhoI for IgL to linearize each vector. PCR products and expression vectors were cloned using In-Fusion Snap Assembly Master Mix (Takara Bio Inc.).

Expression plasmids were obtained by transforming of Stellar Competent Cells (Takara Bio) and purified using the QIAprep Spin Miniprep Kit (QIAGEN). To screen for reactivity to integrin α_v_β_6_, antibodies were produced in HEK293 cells (originally obtained from ATCC, CRL-1573) by transfection with Lipofectamine 3000 (Thermo Fisher Scientific). Transfected cells were maintained in DMEM containing 2% FBS for 5 days, and then the supernatant was used for ELISA. Plasmid DNA from reactive clones was transformed into Stellar Competent Cells and purified using the QIAGEN Plasmid Maxi Kit.

### Antibody production.

Recombinant antibodies were transiently expressed using the ExpiCHO Expression System (Thermo Fisher Scientific). ExpiCHO cells were cotransfected with a mixture of expression vectors for the heavy and light chains of the antibodies following the manufacturer’s protocol.

After 10 days of culturing of the transfected cells, the clarified culture supernatant was loaded into Ab-Capcher (ProteNova), and mAbs were purified in accordance with the manufacturer’s instructions. Disposable plastic columns (Thermo Fisher Scientific) were used according to the manufacturer’s recommended protocol to obtain solubilized recombinant mAbs.

### Preparation of human IgG.

Ab-Rapid SPiN (P-013, ProteNova) was used to purify IgG from the sera of patients with UC and controls. The purified IgG was then dialyzed against PBS (pH 7.2), and concentrated by ultrafiltration with an Amicon Ultrafilter (UFC805024, Millipore) followed by storage at –20°C. Purified IgG concentrations were measured using a NanoDrop 2000c spectrophotometer (Thermo Fisher Scientific).

### ELISA.

ELISA starter accessory kits (E101, Bethyl Laboratories) were used in accordance with the instructions of the manufacturer. Microtiter plates were coated using carbonate-bicarbonate buffer (coating buffer) with 100 μL of 2 μg/mL recombinant human integrin α_v_β_6_ heterodimer proteins (IT6-H52E1, ACROBiosystems), incubated overnight at 4°C, washed 3 times with TBS containing 0.05% Tween 20 (wash solution), and blocked with TBS containing 1% BSA for 30 minutes at approximately 25°C. After 3 washes with wash solution, 10-fold serial dilutions of mAbs starting at 10 μg/mL and 3 μg/mL (diluted with TBS with 0.05% Tween 20 and 1% BSA) were added, and plates were incubated for 1 hour at approximately 25°C. Plates were washed 5 times and incubated with 100 μL goat anti-human IgG antibody conjugated with horseradish peroxidase (1:50,000; ab6759, Abcam) at 25°C for 60 minutes. After washing, the bound antibodies were detected by incubation with 3,30,5,50-tetramethylbenzidine for 10 minutes. Absorbance was noted at 450 nm. EC_50_ values were calculated by nonlinear regression analysis on the binding curves using GraphPad Prism version 10 (GraphPad Software). ELISA was carried out in the presence or absence of MgCl_2_ and CaCl_2_ (1 mM each). MgCl_2_ and CaCl_2_ were added to buffer for washing, blocking, and dilution of antibodies. The same method was used to assess reactivity with other integrins. The mAb 10D5 (ab77906, Abcam) was used as the positive control and IgG derived from healthy individuals (143-09501, Fujifilm Wako Pure Chemical) as the negative control.

To examine whether the RGD (Arg-Gly-Asp) peptide blocked the binding of each mAb to integrin α_v_β_6_, the RGDS (Arg-Gly-Asp-Ser) peptide (A9041, Sigma-Aldrich) or the control RGES (Arg-Gly-Glu-Ser) peptide (A5686, Sigma-Aldrich) was added to each mAb before incubation. In experiments with peptides, the final concentration of mAb was 3 μg/mL, and each peptide was adjusted to 5 concentrations: 0 μg/mL, 12.5 μg/mL, 25 μg/mL, 50 μg/mL, and 100 μg/mL.

We used an anti–integrin α_v_β_6_ ELISA kit (catalog 5288, Medical & Biological Laboratories) for detecting anti–integrin α_v_β_6_ IgG antibody titers from patients with UC according to the manufacturer’s instructions.

### Biolayer interferometry.

The affinity between each mAb and integrin α_v_β_6_ was measured using biolayer interferometry (BLI) with an Octet RED96 (ForteBio). Biotinylated integrin α_v_β_6_ (IT6-H82E4, ACROBiosystems) was loaded at 25 nM in kinetics buffer (0.1% BSA, 0.6 M sucrose, 0.02% Tween 20, 1 mM MgCl_2_, and CaCl_2_ in TBS) for 300 seconds onto a SAX2 biosensor (ForteBio). The association of integrin α_v_β_6_ and mAbs at 200, 50, 12.5, 3.13, and 0.78 nM was measured in kinetics buffer for 300 seconds. The measurement range was adjusted according to the dissociation constant (K_D_) value of each mAb. Dissociation in kinetics buffer was measured for 300 seconds. The on-rate constant (K_on_), off-rate constant (K_off_), and K_D_ values were calculated using a global fit to a 1:1 binding model.

### Solid-phase integrin α_v_β_6_ binding assay.

For this assay, 96-well microtiter plates were previously coated using coating buffer with either 5 μg/mL of fibronectin (F0985, Sigma-Aldrich) or 0.5 μg/mL of LAP (LAP-H5213, ACROBiosystems) (100 μL/well, 4°C, overnight). After removal of the coating solution, the plates were blocked by TBS containing 1% BSA. In another 96-well plate, 60 μL/well of a 2× stock (4 μg/mL of integrin α_v_β_6_ with His-tag for fibronectin or 0.4 μg/mL integrin α_v_β_6_ with His-tag for LAP) was combined with 60 μL/well of a 2× stock of each mAb diluted in the same way as for ELISA and incubated for 1 hour. After the ligand-coated plates were washed, 100 μL of the mAb–integrin α_v_β_6_ mixture was transferred to the ligand-coated plate and incubated for 1 hour. After washing of the plate with wash solution, an Anti-His-tag mAb-HRP-DirecT (1:5,000; D291-7, Medical & Biological Laboratories) was added followed by incubation for 60 minutes. After the wash, bound antibodies were incubated with 3,30,5,50-tetramethylbenzidine for 10 minutes for detection. The absorbance was measured at 450 nm.

### Statistics.

Statistical analysis was conducted using GraphPad Prism (version 10). The correlation between anti–integrin α_v_β_6_ IgG titers and blocking activity of integrin α_v_β_6_–fibronectin or integrin α_v_β_6_–LAP binding was evaluated using the Pearson product-moment correlation. A *P* value less than 0.05 was considered to indicate statistical significance. For experiments using patient-derived IgG, the cutoff OD values for the antibody titer and the inhibitory effect were defined as the mean value of control IgG plus 3 SD.

### Study approval.

The study was conducted in accordance with the Declaration of Helsinki and was approved by the Ethics Committee of the Graduate School of Medicine, Kyoto University, Kyoto, Japan (protocol R1004). Written informed consent was obtained from all patients after they were provided with a full explanation of the nature and possible outcomes of the study. Recombinant DNA experiments were performed following approval by the Kyoto University Recombinant DNA Experiment Safety Committee (approval 230103).

### Data availability.

Values for all data points shown in graphs and values behind any reported means are provided in the Supporting Data Values XLS file accompanying this article. No next-generation sequencing data were generated in this study. All BCR variable region sequences were obtained by Sanger sequencing, which does not fall under the MINSEQE guidelines. All data supporting the findings of this study are included in the article or its supplemental materials. Additional information is available upon reasonable request.

## Author contributions

IT, YN, MS, and TK conceptualized the study. IT and YN developed methodology. IT and YN performed formal analysis. IT, YN, and DJCJ performed investigation. MS acquired funding. IT and YN provided resources. TC and HS supervised the study. IT performed visualization. IT and YN prepared the original draft of the manuscript. MS, TK, SO, TY, KS, AH, M Yasuda, KC, RN, M Yokode, YM, SM, TM, TC, and HS reviewed and edited the manuscript.

## Funding support

- Japan Society for the Promotion of Science KAKENHI grant 24K02437 (to MS).
- Japan Agency for Medical Research and Development (23ek0109597 to MS).

## Supplementary Material

Supplemental data

Supporting data values

## Figures and Tables

**Table 1:**
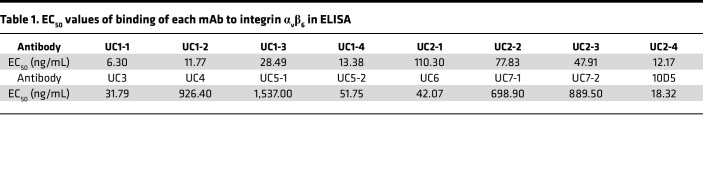
EC_50_ values of binding of each mAb to integrin α_v_β_6_ in ELISA

**Table 2:**
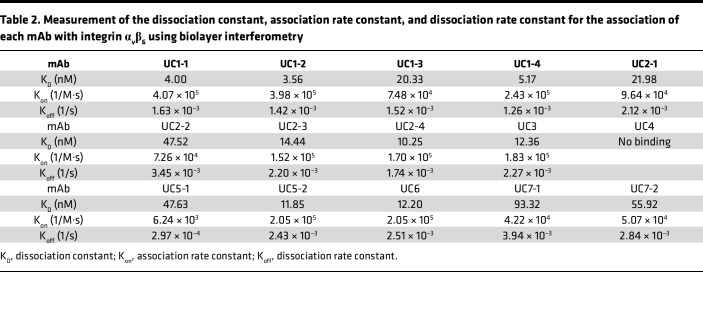
Measurement of the dissociation constant, association rate constant, and dissociation rate constant for the association of each mAb with integrin α_v_β_6_ using biolayer interferometry

**Table 3:**
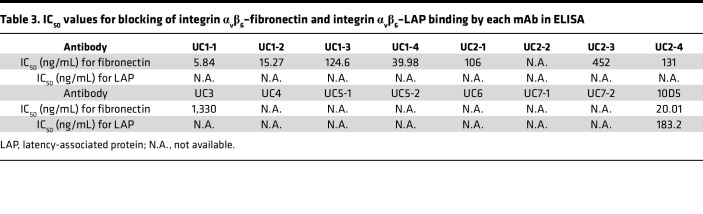
IC_50_ values for blocking of integrin α_v_β_6_–fibronectin and integrin α_v_β_6_–LAP binding by each mAb in ELISA

## Version 1 01/08/2026

In-Press Preview

## Version 2 02/23/2026

Electronic publication
