# Supplementary material for: Characteristics of anti–integrin αvβ6 autoantibodies in patients with ulcerative colitis
Source: JCI Insight. 2026 Jan 8;11(4):e192953. doi: 10.1172/jci.insight.192953 (PMC12956020; doi:10.1172/jci.insight.192953)
Supplement: Supplemental data [file jciinsight-11-192953-s126.pdf]

Supplementary Material

Supplementary Figures:

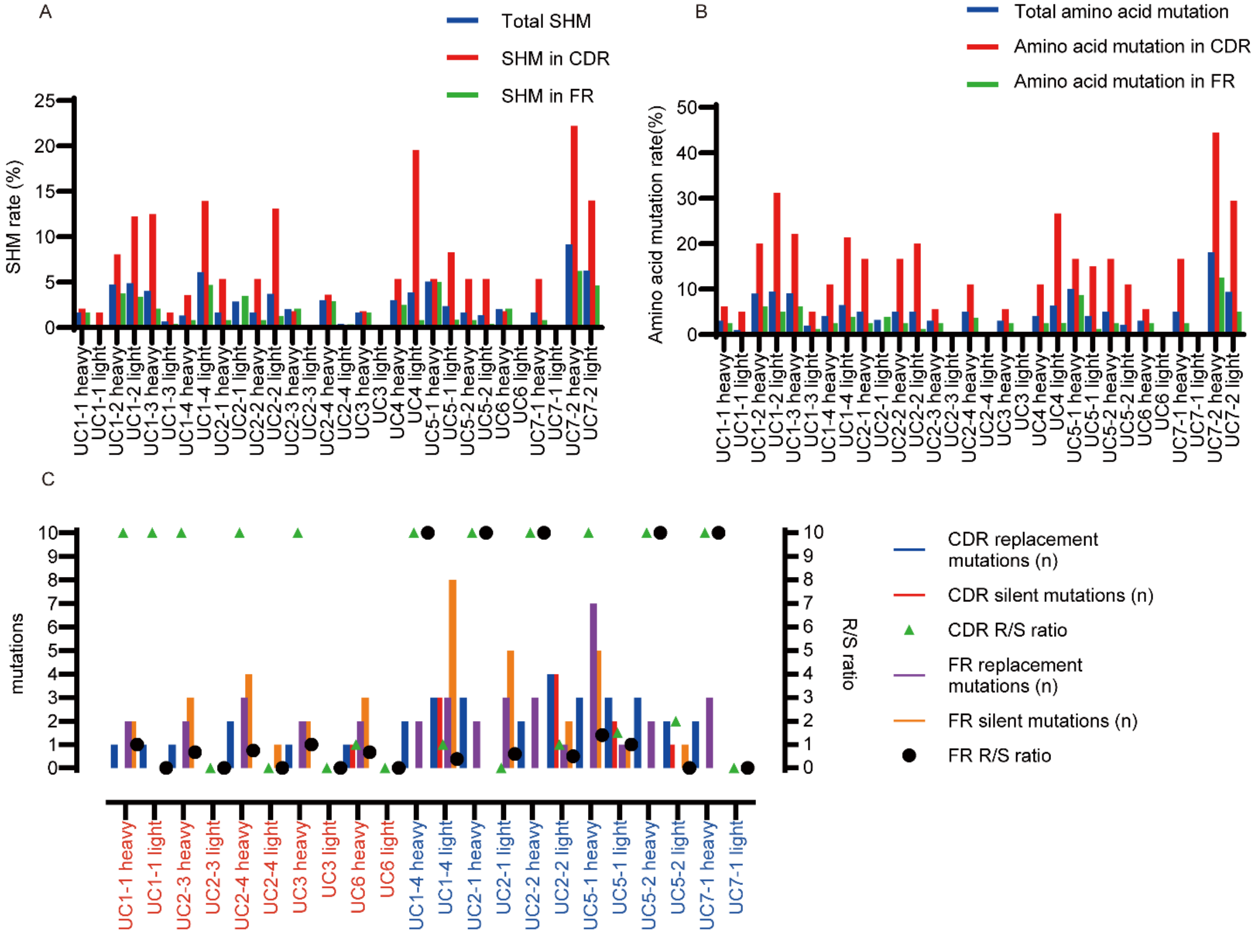

Supplemental Figure 1. Analysis of SHM and amino acid-level alterations in patient-derived mAbs

Mutation rates were calculated only within the V gene region (FR1-3, CDR1, 2, and the V-gene-encoded portion of CDR3).

(A) Nucleotide-level SHM rates across the variable regions of the heavy and light chains. **(B)** Frequencies of amino acid substitutions across the variable regions of the heavy and light chains. **(C)** The number of replacement and silent mutations in the CDR and FR regions of the heavy chain was quantified. Antibodies were grouped based on the presence of either CDR-H1 or CDR-H2. R/S ratios were calculated; values with no silent mutations (R/S = ∞) were plotted at 10 for visualization.

SHM, somatic hypermutation; mAb, monoclonal antibody; CDR, complementarity-determining region; R/S, replacement/silent

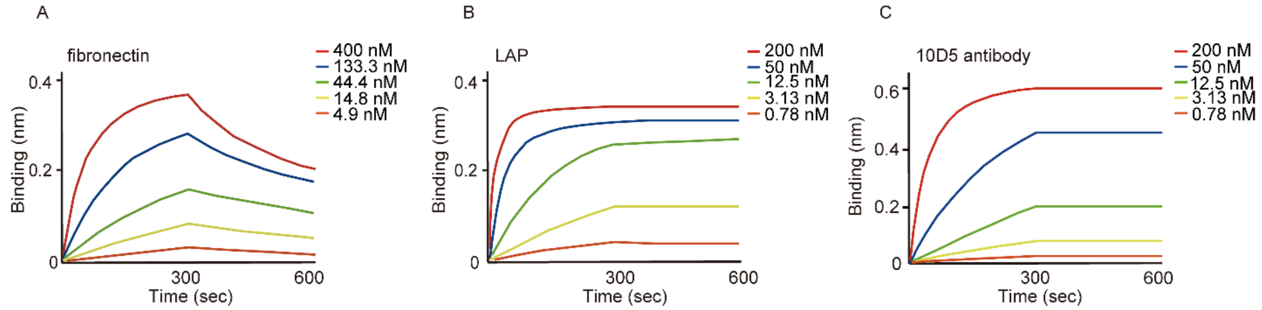

Supplemental Figure 2. Evaluation of affinity of each mAb for integrin αvβ6 using BLI

(A) BLI was performed in the presence of cations in the buffer to confirm the affinity of each mAb for integrin αvβ6. The measurement range was optimized according to the KD value of each mAb. Most mAbs had adequate affinity for integrin αvβ6, similar to that noted in ELISA; however, some mAbs with high EC50 concentrations (UC5-1, UC7-1, and UC7-2) in ELISA showed low affinity in BLI, and UC4 did not bind sufficiently in BLI.

(B and C) BLI measurements were performed in the absence of cations using both UC1-1(B) and UC4 (C). UC1-1 antibody did not react with integrin αvβ6, consistent with the results noted in ELISA. UC4 antibody also did not react with integrin αvβ6, contrary to ELISA results.

mAb, monoclonal antibody; BLI, biolayer interferometry; KD, dissociation constant; ELISA, enzyme-linked immunosorbent assay; EC50, half-maximum effective concentration

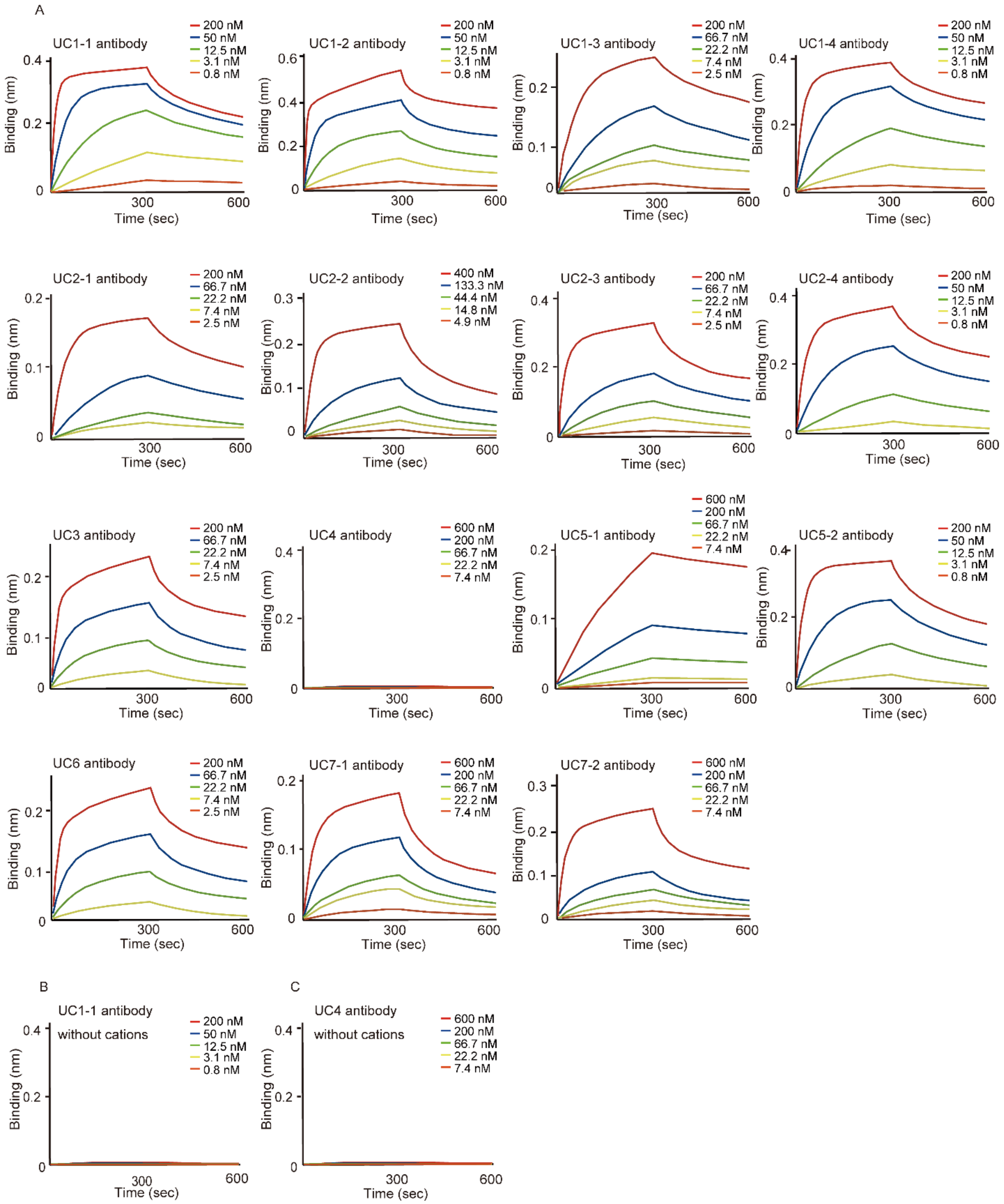

Supplemental Figure 3. Evaluation of affinity of fibronectin, LAP, and 10D5 antibody to integrin αvβ6 using BLI

(A to C) BLI sensorgrams of fibronectin (A), LAP (B), or 10D5 antibody (C) binding to integrin αvβ6 are shown. The analysis revealed that while fibronectin showed measurable binding kinetics

(KD = 47.18 nM), LAP and 10D5 antibody demonstrated extremely stable binding with negligible dissociation (Koff< 1.0 × 10^{-7} 1/s).

BLI, biolayer interferometry; LAP, latency-associated protein; KD, dissociation constant; Koff, dissociation rate constant

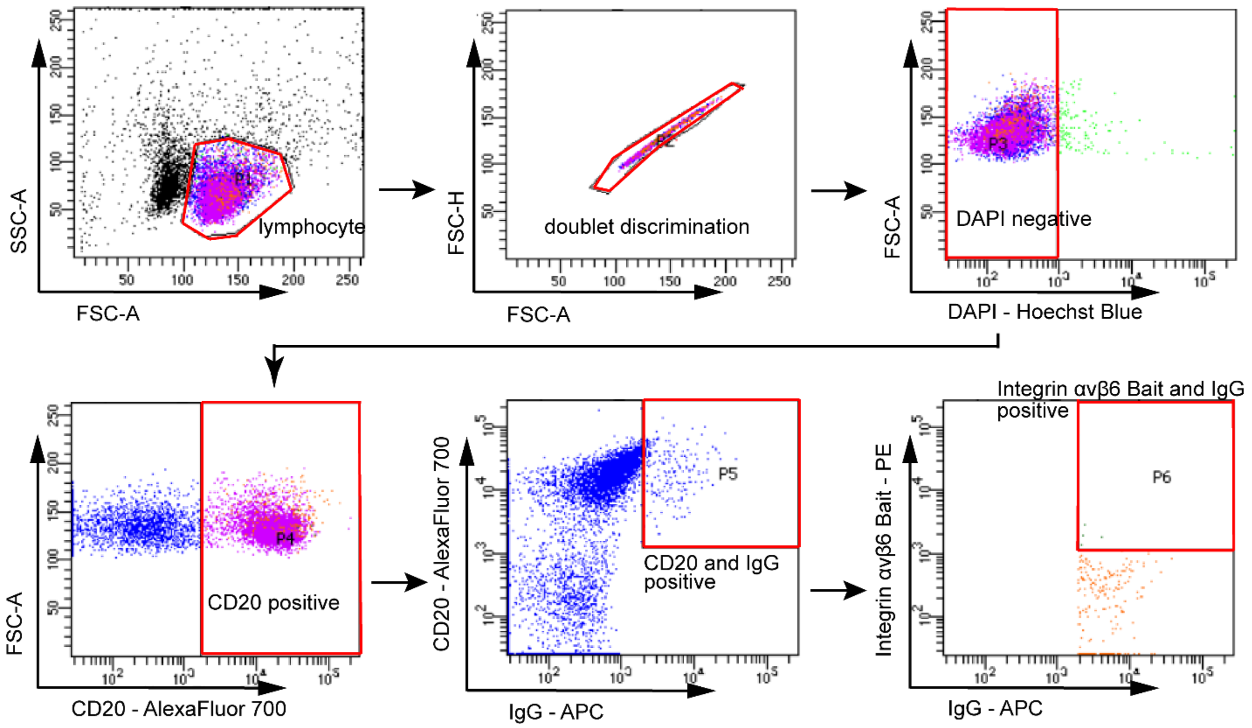

Supplemental Figure 4. Representative gating strategy for the isolation of integrin αvβ6-specific IgG⁺ B cells from PBMCs or lymph node cells.

Lymphocytes were first gated based on side and forward scatter characteristics (SSC-A vs FSC-A), followed by doublet discrimination using FSC-H vs. FSC-A. Viable cells were identified by excluding $DAPI^+$ (Hoechst $blue^+$) cells. $CD20^+$ B cells were selected based on FSC-A and CD20-AlexaFluor700 expression. $CD20^+IgG^+$ B cells were then identified using CD20 and IgG staining. Finally, integrin αvβ6-specific cells were gated by selecting double-positive cells for IgG and biotinylated integrin αvβ6 with PE-labeled NeutrAvidin (Integrin αvβ6 bait). A representative result is shown from lymph node cells of P7.

PBMCs, peripheral blood mononuclear cells; FSC-H, forward scatter-height; FSC-A, forward scatter-area

Supplementary Tables:
Supplementary Table 1. Patients' clinical data

Patient ID	Age, years	Sex	Collection date	Sample source	Age of diagnosis	Extent of disease	Total Mayo score	MES	Extraintestinal complication	Therapy	Operation	Causes leading to surgery
UC1	63	Female	2020/9/9	PBMC	59	pancolitis	4	1	deep vein thrombosis	tacrolimus, mercaptopurine hydrate	-	
UC2	49	Male	2022/2/20	lymph nodes	44	pancolitis	10	3	-	5ASA, tacrolimus, Infliximab	+	Resistance to medical treatment
UC3	49	Female	2022/3/31	lymph nodes	27	proctitis	3	1	-	none	+	dysplasia
UC4	68	Male	2022/8/4	lymph nodes	61	pancolitis	5	2	-	none	+	dysplasia
UC5	54	Female	2023/2/13	PBMC	35	left-sided colitis	5	2	-	SASP	-	
UC6	52	Male	2023/3/22	PBMC	41	pancolitis	8	3	-	5ASA	-	
UC7	39	Male	2023/7/3	lymph nodes	19	pancolitis	5	3	-	none	+	dysplasia

PBMC, peripheral blood mononuclear cells; MES, Mayo Endoscopic Score; 5ASA, 5-aminosalicylic acid; SASP, salazosulfapyridine

Supplementary Table 2. Somatic hypermutation of each mAb

mAb ID	chain	total SHM * (%)	SHM in CDR ** (%)	SHM in FR *** (%)	Total amino acid mutation (%)	Amino acid mutation in CDR ** (%)	Amino acid mutation in FR *** (%)	CDR replace-ment mutations (n)	CDR silent mutations (n)	CDR R/S ratio	FR replace-ment mutations (n)	FR silent mutations (n)	FR R/S ratio
UC1-1	heavy	1.69	2.08	1.67	3.06	6.25	2.50	1	0	∞ ****	2	2	1.00
	kappa	0.33	1.67	0.00	1.01	5.00	0.00	1	0	∞	0	0	0.00
UC1-2	heavy	4.75	8.06	3.75	9.00	20.00	6.25	1	0	∞	5	4	1.25
	kappa	4.90	12.24	3.37	9.47	31.25	5.06	5	1	5.00	4	4	1.00
UC1-3	heavy	4.05	12.5	2.08	9.09	22.2	6.25	4	3	1.33	5	0	∞
	lambda	0.68	1.64	0.43	2.04	5.00	1.28	1	0	∞	1	0	∞
UC1-4	heavy	1.35	3.57	0.83	4.04	11.11	2.50	2	0	∞	2	0	∞
	lambda	6.13	13.95	4.70	6.52	21.43	3.85	3	3	1.00	3	8	0.38
UC2-1	heavy	1.69	5.36	0.83	5.05	16.67	2.50	3	0	∞	2	0	∞
	lambda	2.87	0.0	3.49	3.25	0.00	3.93	0	0	0.00	3	5	0.60
UC2-2	heavy	1.69	5.36	0.83	5.05	16.67	2.50	2	0	∞	3	0	∞
	lambda	3.72	13.11	1.28	5.10	20	1.28	4	4	1.00	1	2	0.50
UC2-3	heavy	2.04	1.81	2.09	3.03	5.56	2.51	1	0	∞	2	3	0.67
	kappa	0.00	0.00	0.00	0.00	0.00	0.00	0	0	0.00	0	0	0.00
UC2-4	heavy	3.05	3.63	2.92	5.05	11.11	3.75	2	0	∞	3	4	0.75
	kappa	0.42	0.00	0.35	0.00	0.00	0.00	0	0	0.00	0	1	0.00
UC3	heavy	1.69	1.82	1.67	3.03	5.56	2.50	1	0	∞	2	2	1.00
	lambda	0.00	0.00	0.00	0.00	0.00	0.00	0	0	0.00	0	0	0.00
UC4	heavy	3.04	5.36	2.50	4.04	11.11	2.50	2	1	2.00	2	4	0.50
	kappa	3.89	19.57	0.84	6.38	26.67	2.53	4	5	0.80	2	0	∞

UC5-1	heavy	5.08	5.36	5.00	10.10	16.67	8.75	3	0	∞	7	5	1.40
	lambda	2.38	8.33	0.85	4.08	15.00	1.28	3	2	1.50	1	1	1.00
UC5-2	heavy	1.69	5.36	0.83	5.05	16.67	2.50	3	0	∞	2	0	∞
	lambda	1.38	5.36	0.43	2.08	11.11	0.00	2	1	2.00	0	1	0.00
UC6	heavy	2.04	1.81	2.09	3.03	5.56	2.51	1	1	1.00	2	3	0.67
	kappa	0.0	0.0	0.0	0.00	0.00	0.00	0	0	0.00	0	0	0.00
UC7-1	heavy	1.69	5.36	0.83	5.05	16.67	2.50	2	0	∞	3	0	∞
	lambda	0.00	0.00	0.00	0.00	0.00	0.00	0	0	0.00	0	0	0.00
UC7-2	heavy	9.18	22.22	6.25	18.18	44.44	12.50	10	5	2.00	8	4	2.00
	kappa	6.27	14.00	4.64	9.37	29.41	5.06	5	2	2.50	4	7	0.57

*Somatic hypermutation in V gene region

**Somatic hypermutation in CDR in V gene region

***Somatic hypermutation in Framework regions 1, 2, and 3

****R/S ratio was considered infinite (∞) when no silent mutations were observed in the analyzed region.

SHM, somatic hypermutation; CDR, CDR, complementarity-determining region; R/S, replacement/silent

Supplementary Table 3. Measurement of KD, Kon, and Koff of fibronectin, LAP, and 10D5 antibody binding to integrin αvβ6 using BLI

	Fibronectin	LAP	10D5
KD (nM)	41.78	not available	not available
Kon (1/ M·s)	4.10×10^{4}	4.25×10^{5}	9.78×10^{4}
Koff (1/s)	1.71×10^{-3}	$<1.0 \times 10^{-7}$	$<1.0 \times 10^{-7}$

LAP, latency-associated protein; KD, dissociation constant; Kon, association rate constant; Koff, dissociation rate constant; BLI, biolayer interferometry

Supplementary Table 4. Results of ELISA using IgG from patients with UC and control patients

Patient ID	Anti-integrin αvβ6 antibody titer (Unit)	Inhibition of fibronectin binding by patient IgG (%)	Inhibition of LAP binding by patient IgG (%)
UC1	280.63	-61.46	8.49
UC2	137.38	58.45	18.05
UC3	11.84	16.45	-0.14
UC4	17.47	59.86	0.84
UC5	2.78	21.83	9.56
UC6	10.22	38.58	2.66
UC7	239.36	92.55	95.17
UC8	6.48	23.19	-12.89
UC10	7.80	20.78	10.68
UC11	4.44	8.71	4.64
UC12	3.00	12.80	-9.36
UC13	12.72	12.62	-5.37
UC14	116.04	56.02	27.41
UC15	7.37	10.60	2.39
UC16	9.13	30.75	-2.04
UC17	20.97	29.08	20.85
UC18	5.88	18.57	-5.83
UC19	20.75	32.20	-3.19
UC20	5.05	23.61	-2.53
UC21	2.78	18.68	-6.61
UC22	10.01	24.55	-1.21
UC23	5.96	28.12	-4.19
UC24	23.03	15.74	24.34
UC26	12.11	36.17	3.14
UC27	3.35	8.50	-5.40
UC28	12.17	27.94	3.30
UC29	84.27	56.60	18.53
UC30	55.19	86.10	7.41
UC31	12.81	46.10	-0.32

UC32	189.30	59.89	93.88
UC33	58.33	77.02	5.96
UC34	31.73	29.08	-3.38
UC35	1653.46	-283.52	84.53
UC36	14.38	15.04	-7.57
UC37	47.30	35.60	41.87
UC38	102.80	45.99	41.41
UC39	204.76	73.78	57.14
UC40	81.49	78.22	22.80
UC41	21.50	53.33	-9.57
UC42	8.35	33.05	-0.99
UC44	16.45	8.94	7.45
UC45	4.62	12.17	8.81
Control15	0.408	2.58	-0.76
Control16	0.058	1.72	7.9
Control19	0.835	3.87	-6.69
Control24	0.743	7.15	-1.84
Control32	1.297	-3.11	0.08
Control48	0.562	11.82	12.77
Control51	0.545	7.78	-4.18
SD for control samples	0.385	4.85	6.88
3SD for control samples	1.154	14.55	20.64

ELISA, enzyme-linked immunosorbent assay; LAP, latency-associated protein; UC, ulcerative colitis
